# Reversion of MRAP2 Protein Sequence Generates a Functional Novel Pharmacological Modulator for MC4R Signaling

**DOI:** 10.3390/biology11060874

**Published:** 2022-06-07

**Authors:** Jing Xu, Meng Wang, Yanbin Fu, Cong Zhang, Zhe Kuang, Shan Bian, Rui Wan, Shen Qu, Chao Zhang

**Affiliations:** 1 Fundamental Research Center, Shanghai YangZhi Rehabilitation Hospital (Shanghai Sunshine Rehabilitation Center), School of Life Sciences and Technology, Tongji University, Shanghai 201619, China; 1911018@tongji.edu.cn (J.X.); ianbin_fu@163.com (Y.F.); zczc0627@163.com (C.Z.); 2001123adc@gmail.com (Z.K.); 2 Department of Plastic and Reconstructive Surgery, Shanghai Institute of Precision Medicine, Shanghai Ninth People’s Hospital, Shanghai Jiao Tong University School of Medicine, Shanghai 200011, China; b21024@sh9hospital.org.cn; 3 Institute for Regenerative Medicine, Shanghai East Hospital, School of Life Sciences and Technology, Tongji University, Shanghai 200120, China; shan_bian@tongji.edu.cn; 4 Department of Critical Care Medicine, Naval Medical Center of PLA, Shanghai 200052, China; 5 Department of Endocrinology and Metabolism, National Metabolic Management Center, Shanghai Tenth People’s Hospital, School of Medicine, Tongji University, Shanghai 200072, China

**Keywords:** MC4R, MRAP2, protein conformation, dimerization, pharmacological modulation

## Abstract

**Simple Summary:**

Reversion of the wild-type protein sequences of single transmembrane melanocortin accessory protein families (MRAP2) in mice and zebrafish created novel functional pharmacological modulators for regulating melanocortin 4 receptor (MC4R) signaling. All of the brand new reversed MRAP2 (rMRAP2) proteins could form proper dimeric topology on the plasma membrane and interact with and affect the ligand-stimulated pharmacological profiles of zebrafish and mouse MC4R signaling in vitro.

**Abstract:**

As a member of the melanocortin receptor family, melanocortin 4 receptor (MC4R) plays a critical role in regulating energy homeostasis and feeding behavior, and has been proven as a promising therapeutic target for treating severe obesity syndrome. Numerous studies have demonstrated that central MC4R signaling is significantly affected by melanocortin receptor accessory protein 2 (MRAP2) in humans, mice and zebrafish. MRAP2 proteins exist as parallel or antiparallel dimers on the plasma membrane, but the structural insight of dual orientations with the pharmacological profiles has not yet been fully studied. Investigation and optimization of the conformational topology of MRAP2 are critical for the development of transmembrane allosteric modulators to treat MC4R-associated disorders. In this study, we synthesized a brand new single transmembrane protein by reversing wild-type mouse and zebrafish MRAP2 sequences and examined their dimerization, interaction and pharmacological activities on mouse and zebrafish MC4R signaling. We showed that the reversed zebrafish MRAPa exhibited an opposite function on modulating zMC4R signaling and the reversed mouse MRAP2 lost the capability for regulating MC4R trafficking but exhibited a novel function for cAMP cascades, despite proper expression and folding. Taken together, our results provided new biochemical insights on the oligomeric states and membrane orientations of MRAP2 proteins, as well as its pharmacological assistance for modulating MC4R signaling.

## 1. Introduction

As seven-transmembrane spanning G protein-coupled receptors (GPCR), the melanocortin receptor (MCR) family consists of five members: MC1R–MC5R. Activation of MCRs leads to adenylate cyclase stimulation and accumulation of the intracellular cyclic adenosine monophosphate (cAMP), with downstream signaling cascades. These five members distribute throughout the body and play distinct roles in regulating physiological processes by selectively binding to various melanocortin peptides [1,2,3,4,5]. Among them, MC4R has been reported to play an indispensable role in energy homeostasis and feeding behavior. Inhibition of MC4R in mice leads to obesity, secondary to a hyperphagic, hyperinsulinemia and hyperglycemic state [6,7]. Therefore, MC4R has become a validated drug target for obesity therapy and several MC4R agonists achieved fabulous outcomes in phase III clinical trials, such as LY2112688 and setmelanotide [8,9,10,11]. MC4R exerts its function by stimulation with a series of endogenous ligands, such as adrenocorticotropin hormone (ACTH) and alpha/beta/gamma-melanocyte stimulating hormone (α/β/γ-MSH), and MC4R shows much lower affinity to γ-MSH than the other three melanocortins [1,12]. In addition to canonically promoting cAMP production, mediated by Gα_s_ upon receiving an agonism signal, MC4R can also recruit β-arrestin to mediate signaling through mitogen-activated protein kinase (MAPK)-mediated phosphorylation of extracellular signal-regulated kinase 1/2 (ERK1/2) [13]. Intriguingly, a recent study demonstrates that the β-arrestin-biased MC4R variants are associated with a lower risk of obesity in humans, suggesting that MC4R plays a key role in regulating body weight through β-arrestin signaling [14].

Melanocortin 2 receptor accessory protein (MRAP) functions as a small protein with a single membrane-spanning domain and conserved N- and C-terminals. It was originally identified as an MC2R chaperone with indispensable roles for the trafficking, ligand binding as well as the signal transduction of MC2R [15]. As a paralogue of MRAP, MRAP2 shares a conserved functional domain and similar structural features with MRAP. However, unlike MRAP, which mainly regulates MC2R in the adrenal gland, MRAP2 shows more preference for the other four MCRs [16,17,18,19,20], as well as several non-melanocortin receptors, such as orexin receptor and ghrelin receptor [21,22,23,24,25]. MRAP2 is involved in energy balance and metabolism by regulating MC4R activity in many organisms [18,20,26,27]. *MRAP2*-deficient mice develop severe obesity, which is presumably resulting from a significant decrease in MC4R sensitivity to α-MSH in the absence of MRAP2 protein [28]. In the *Xenopus laevis* and *tropicalis* (African clawed frog), MRAP and MRAP2 both exert potentiation patterns on modulating MC3R and MC4R signaling, stimulated by α-MSH and ACTH [16,29,30]. In the *Danio rerio* (zebrafish), Mrap2 exists in two isoforms, Mrap2a and Mrap2b. They express at different time points over the course of embryonic development and exhibit distinct functions on modulating Mc4r signaling. Simply, Mrap2a mainly expresses in larvae and facilitates zebrafish growth by specifically blocking the action of Mc4r, while Mrap2b expresses in a later developmental stage and increases the pharmacological sensitivity of Mc4r to the endogenous ligand [31,32].

Previous studies found that mammalian MRAP2 existed on cell membranes as parallel or antiparallel homodimers, and it could also form higher-order oligomers [22,33,34]. Similarly, zebrafish Mrap2a and Mrap2b also parallelly or antiparallelly dimerize with themselves or with each other [35]. When the orientation of the Mrap2a and Mrap2b dimers was artificially fixed, the antiparallel Mrap2b homodimer was more similar to wild-type Mrap2b, in terms of pharmacological effects on MC4R, suggesting that Mrap2b may exist primarily as antiparallel homodimers [36]. The presence of MRAP2 antiparallel dimers suggests that the N-terminus of MRAP2 orientates both intracellularly and extracellularly, but the difference between these two orientations, and whether it is a random event or one of the orientations dominates, remains unclear. Therefore, we put forward a hypothesis that the two orientations of MRAP2 are functionally different, and the reversion of MRAP2 will not affect the conformation but may influence its function in assisting MC4R signaling. In this study, we synthesized fully reversed MRAP2s (RMRAP2s), whose protein sequence is opposite to the wild type, and assessed their capability to interact with MC4R, dimerization and the pharmacological modulation on MC4R signaling. We demonstrated that RMRAP2s showed similar conformation with wild-type ones and found that the dual orientations of MRAP2s were not a random event. In addition, Mrap2a and RMrap2a exhibited an opposite pharmacological effect on Mc4r signaling, while Mrap2b and RMrap2b similarly regulated Mc4r cascades. However, the reversion of mouse MRAP2 obviously disrupted its function for regulating MC4R trafficking. Overall, these findings elucidated the complex intrinsic topology of single transmembrane proteins and may provide scientific inspiration on how to create or optimize novel transmembrane accessory and allosteric modulators for melanocortin receptors, or other GPCRs, in the future.

## 2. Materials and Methods

### 2.1. Multiple Sequence Alignment and TM Prediction

**Figure 1:**
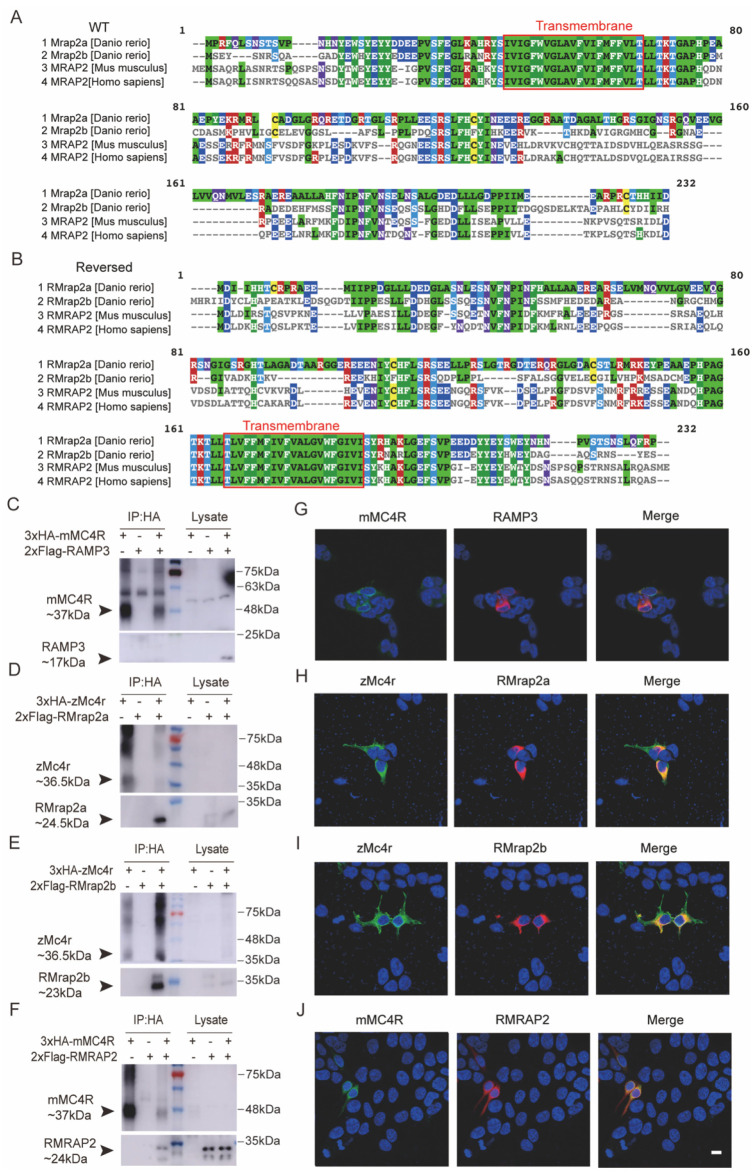
Interaction of RMRAP2 and RMrap2a/b with mMC4R and zMc4r. (**A**,**B**) Multiple sequence alignment by MUSCLE (3.8) of wild (**A**) and reversed (**B**) zebrafish Mrap2a/b, mouse MRAP2 and human MRAP2. Red frame indicates the transmembrane region. (**C**,**G**) Negative control. Mouse RAMP3 did not interact with MC4R. (**D**–**F**) Interactions of MRAP2 with MC4R proteins. Coimmunoprecipitation of 3xHA-Mc4r with 2xFlag-RMrap2a (**D**) or 2xFlag-RMrap2b (**E**) or mouse MC4R and RMRAP2 (**F**) in HEK293T cells. MC4R was detected with mouse anti-HA antibody; MRAP2 was detected with mouse anti-Flag antibody. IP: protein samples with anti-HA immunoprecipitation. Lysate: relevant protein samples to the samples in the IP group but without any immunoprecipitation. (**G**–**J**) Corresponding immunofluorescence of co-localization of protein complexes on the cell surface. Green indicates zMc4r or mMC4R, and red indicates RMRAP2s. Scale bar = 10 μm. mMC4R: mouse MC4R, zMc4r: zebrafish Mc4r, RMrap2a: reversed zebrafish mrap2a, RMrap2b: reversed zebrafish mrap2b, mRMRAP2: mouse MC4R. The uncropped Western blot figures were presented in Figure S2.

The multi-sequence alignment shown in Figure 1 was performed by MUSCLE. Available online: https://www.ebi.ac.uk/Tools/msa/muscle/ (accessed on 26 June 2021). The alignment results were presented with MView. Available online: https://www.ebi.ac.uk/Tools/msa/mview/ (accessed on 26 June 2021), where amino acids were labeled by different colors, according to their conservative property. TM prediction was accomplished by TMHMM Server v. 2.0. Available online: http://www.cbs.dtu.dk/services/TMHMM/ (accessed on 18 December 2021).

### 2.2. Plasmids

Zebrafish *mc4r* and *mrap2a/b*, and mouse *Mc4r* and *Mrap2* were cloned from zebrafish and mouse brain cDNA, respectively. Reversed *mrap2a/b (Rmrap2a/b)* and reversed *Mrap2 (RMrap2)* were synthesized from GENEWIZ company (Suzhou, China). All the genes above were inserted in the pcDNA3.1(+/−) vector, with 3xHA or 2xflag tag added at the 5′-end. For the bimolecular fluorescent complimentary (BiFC) assay, the YFP-F1/F2 sequence was, respectively, fused to the 3′-end or 5′-end of *Rmrap2a/b* and *RMrap2*.

### 2.3. Cell Culture and Transfection

Human embryonic kidney (HEK) 293T cells were cultured in Dulbecco’s Modified Eagle’s Medium (DMEM) (HyClone, Logan, UT, USA, SH30243.01), containing 10% fetal bovine serum (FBS) and 1% penicillin/streptomycin, at 37 °C with 5% CO_2_. Transfection was carried out with polyethylenimine L (PEI) (Thermo Fisher, Waltham, MA, USA, BMS1003-A) until cells grew to 70–80% confluency. The indicated plasmids were mixed in serum-free DMEM and PEI solution was added to the plasmid mixture at a 4:1 ratio, and then it was gently pipetted to mix the solution. Finally, the mixture was applied into cells for 15 min incubation.

### 2.4. Western Blot and Co-Immunoprecipitation (CoIP)

Between 24–48 h after transfection (*Mc4r:RMrap2* = 1:1 or *Mrap2:Rmrap2* = 1:1, and the DNA total amount was kept at 2 µg for each well in 6-well plates), DMEM was removed and 400 µL of IP cell lysis buffer (Beyotime, Shanghai, China, P0013) was added to each well. Next, the cell lysis was transferred to 1.5 mL tubes and rotated for 1 h at 4 °C. The cells were then centrifuged for 15 min at 4 °C and 17,000× *g*. The supernatant was transferred to a new tube. A total of 40 µL of the supernatant was retained as a control and the rest of the specimen was incubated with anti-HA-Mouse (Cell Signaling Technology, Inc., Boston, MA, USA, 2367S) overnight at 4 °C. Total protein was stored at −20 °C upon the addition of protein loading buffer. The next day, Protein A+G Agarose (Beyotime, Shanghai, China, P2055) beads were added to the IP tubes and rotated for 4 h at 4 °C. The beads were then washed three times with cell lysis buffer containing protease inhibitor and centrifuged at 4 °C and 1000× *g* to discard the supernatant. Finally, 40 µL of 1× protein loading buffer was added to resuspend the beads. Both the total and the IP protein were boiled at 95 °C for 15 min. For SDS-PAGE, 12% gel was utilized and 10 µL of each sample was loaded. HA-Rabbit (1:5000) (Cell Signaling Technology, Inc. 3724S) and DYKDDDDK (Flag)-Rabbit (1:5000) (Cell Signaling Technology, Inc. 14793S) antibodies were utilized to blot MC4R and RMRAP2 or other proteins, respectively.

### 2.5. Immunofluorescence Assay

Cells were seeded in Poly-D-lysine pre-treated 12-well plates with slides and transfected with the indicated plasmids (*Mc4r:RMrap2* = 1:1 or *Mrap2:RMrap2* = 1:1, and the DNA total amount was kept at 1µg for each well in the 12-well plates). Between 24–48 h later, the media was removed, and 4% paraformaldehyde was added to fix the cells. Anti-HA and anti-Flag antibodies (1:1000) were applied to incubate the cells for 2 h at room temperature, and then, the cells were washed three times with DPBS (Beyotime, Shanghai, China, C0021G). Alexa Fluor488 and 647 IgG (Abcam, Cambridge, UK, ab150077 and ab150083) were employed as secondary antibodies for HA and Flag, respectively. After incubation with fluorescent secondary antibodies for 2 h, protected from light, the cells were washed three times with PBST. Next, ProLong(R) Gold Antifade with DAPI Molecular Probes (Cell Signaling Technology, Inc., 8961SDAPI) was applied on the glass slides and covered by transparent slides. Finally, the slides were observed with a Carl Zeiss confocal laser scanning microscope under a 63x oil lens.

### 2.6. Bimolecular Fluorescent Complimentary (BiFC) Assay

Complementary YFP-F1 and YFP-F2 fragments were constructed in the C-terminal or N-terminal of MRAP2 or RMRAP2. The cells were seeded in Poly-D-lysine pre-treated 12-well plates with slides and transfected with the desired plasmids containing YFP-F1 or YFP-F2 (1:1, the DNA total amount was kept at 1µg for each well in the 12-well plates) the following day. Between 24–48 h later, the media was removed and 4% paraformaldehyde was added to fix the cells. The cells were then washed three times with DPBS and ProLong(R) Gold Antifade with DAPI Molecular Probes was applied on glass slides and covered by transparent slides. Finally, the slides were observed with a Carl Zeiss confocal laser scanning microscope under a 63× oil lens.

### 2.7. ELISA Assay

The cells were seeded in Poly-D-lysine pre-treated 24-well plates and transfected with 3xHA*-mMc4r* and 2xFlag-*RMrap2* (or homologous zebrafish genes) at ratio 1:0, 1:3 and 1:6 (the DNA total amount was kept at 0.25 µg for each well in the 24-well plates). The amount of *Mc4r* was fixed and the rest was complemented with blank pcDNA3.1 vector. Between 24–48 h later, the DMEM was removed and 4% paraformaldehyde was added to fix the cells. After blocking with 5% milk in PBS, the cells were incubated with anti-HA antibody (1:2000) for 2.5 h at room temperature and washed three times with DPBS. Anti-IgG secondary antibody (Cell Signaling Technology, Inc. 91196S) was added to each well and incubated for 2 h at room temperature, and then the cells were washed three times with DPBS. Next, the cells were incubated with tetramethylbenzidine (TMB) substrate (Beyotime, Shanghai, China, P0209-500 mL) for 15–30 min at room temperature, protected from light. Finally, the supernatant was transferred to a clear 96-well plate containing 2 M sulfuric acid and absorbance was measured with an ELISA reader (SpectraMax iD3) at OD = 450 nm. Janus Green was utilized to normalize the cell numbers.

### 2.8. cAMP Luminescent Assay

The cells were seeded in 24-well plates and transfected with a mixture of Renilla (to normalize cell numbers), Pcre-luc (to measure cAMP induced by Gs), *mMc4r* and *RMrap2* (or zebrafish homologous genes). The DNA total amount was kept at 0.25 µg for each well in the 24-well plates. The amount of Mc4r was fixed and the rest was complemented with blank pcDNA3.1 vector. Between 24–48 h later, the medium was replaced by fresh DMEM containing 0.1% bovine serum albumin (BSA) as well asα-MSH, ranging from 10^−12^ M to 10^−6^ M. After a 4 h incubation, the Dual-Glo Luciferase Assay System (Promega, Madison, WI, USA, E2940) was applied to measure the cAMP level, according to the manufacturer’s instructions. The cell mixture was transferred to white 96-well plates and the firefly luminescent intensity was measured on the ELISA reader (SpectraMax iD3) with the Dual-Glo luciferase protocol. The Renilla was measured to normalize the cell numbers upon the addition of stop solution. For antagonism experiments, the cells were stimulated by 1 × 10^−8^ M α-MSH for zebrafish and 1 × 10^−7^ M α-MSH for mice, with a concentration of SHU9119 ranging from 10^−12^ M to 10^−6^ M. Three biological replicates (independent transfections) were performed for each group.

### 2.9. Statistical Analysis

All the experiments were conducted at least three times. Data were analyzed using GraphPad Prism 8 (GraphPad Software, Inc., San Diego, CA, USA). cAMP assay results were analyzed by nonlinear regression (curve fit). The results of the surface ELISA were analyzed by a one-way ANOVA. All the results were shown as mean ± SEM. ns (not significant), * *p* < 0.05, ** *p* < 0.01, *** *p* < 0.001 and **** *p* < 0.0001.

## 3. Results

### 3.1. Interaction of Mouse and Zebrafish RMRAP2s with MC4Rs

We selected mouse MRAP2 (mMRAP2, ~24 kDa) and zebrafish Mrap2a/b (zMrap2a/b) and artificially constructed reversed MRAP2 protein sequences (Figure 1 and Figure S1). Multi-sequence alignment showed the most conservative sequence at the N-terminal and within the transmembrane (TM) domain in wild-type (WT) MRAP2 (Figure 1A). Reversed MRAP2s (RMrap2a/b, RMRAP2) shared the same amino acids with WT ones but owned fully reversed orientation of both amino and carboxyl terminals (Figure 1B). Like mouse MRAP2, zebrafish Mrap2a (~24.5 kDa) and Mrap2b (~23 kDa) both interacted with zMc4r (zMc4r, ~36.5 kDa), respectively [28,31]. We therefore focused on the pharmacological role of MRAP2s in modulating MC4R signaling to uncover whether RMRAP2s exhibited a similar function to wild-type MRAP2s. First, 2xFlag tagged reversed *mrap2a/b* and 3xHA-*mc4r* were transfected into HEK293T cells and co-immunoprecipitation (CoIP) assays found that RMrap2a/b interacted with zMc4r (Figure 1D,E) and that RMRAP2 also formed a protein complex with mMC4R (−37 kDa) (Figure 1F). Receptor activity modifying protein 3 (RAMP3, ~17 kDa) was utilized as a negative control (Figure 1C,G) because RAMP3 functioned as a single transmembrane protein like MRAP2, but it could not interact with MC4R [37]. Some dispersive bands ranging from 60 to 80 kDa were seen on the Western blot, indicating the existence of MC4R polymers (Figure 1C–F). Next, immunofluorescence was employed to visualize RMrap2a/b-zMc4r or RMRAP2-mMC4R complexes. Merged fluorescence indicated the co-localization of RMRAP2s and MC4Rs on the cell surface (Figure 1F–H), further confirming the direct interaction of RMRAP2s and MC4Rs both in mice and zebrafish. Overall, our results demonstrated that RMRAP2s could express normally and function as MC4R partners in live cell membranes.

### 3.2. RMRAP2s Could Form Homodimers and Heterodimers

**Figure 2:**
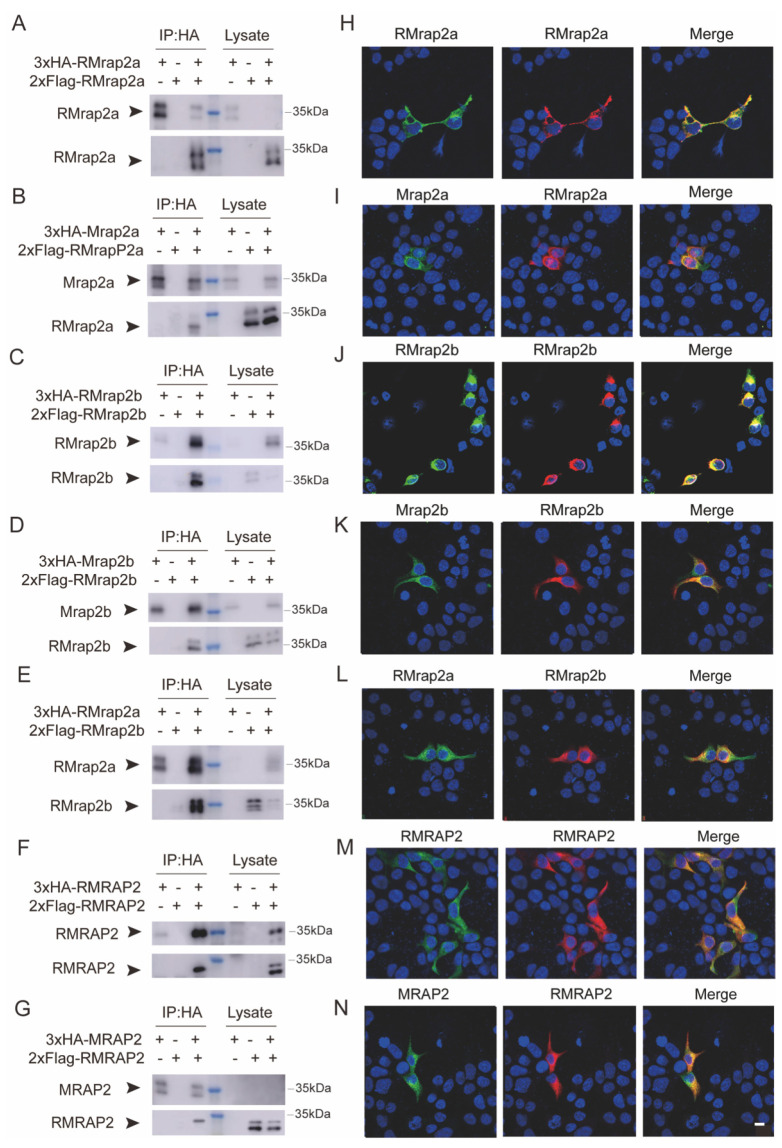
Formation of homodimers and heterodimers of RMRAP2 and RMrap2a/b. (**A**,**B**) The dimerization of RMrap2a with itself (**A**) or with Mrap2a (**B**). The blue marker indicates approximate Mol. wt. of RMrap2a and Mrap2a. (**C**,**D**) The dimerization of RMrap2b with itself (**C**) or with Mrap2b (**D**). (**E**) Co-immunofluorescence of RMrap2a and RMrap2b. (**F**,**G**) The dimerization of mouse RMRAP2 with itself (**J**) or with MRAP2 (**K**). (**H**–**N**) Immunofluorescence of the co-localization of the CoIP protein complexes on the plasma membrane. Scale bar = 10 μm. The uncropped Western blot figures were presented in Figure S2.

In our previous study, we found that Mrap2a and Mrap2b could not only form parallel and antiparallel homodimers on plasma membranes but also form parallel and antiparallel heterodimers with each other [36]. To test the potential dimeric topology of RMrap2a/b, we performed CoIP and immunofluorescence assays with the modified plasmids. As shown in Figure 2, RMrap2a could interact with itself (Figure 2A,H). Similar results were obtained for both RMrap2b and RMRAP2 (Figure 2C,F,J,M). In addition, like wild-type ones, RMrap2a and RMrap2b interacted with each other (Figure 2E,L), suggesting that the RMRAPs were structurally like WTs, and the reversion of the protein sequence did not disrupt the dimeric interplay of each monomer. Moreover, we examined the interplay of wild-type and reversed MRAP2 proteins. As expected, all three RMRAP2s interacted with the corresponding WT ones (Figure 2B,D,G,I,K,N). These results validated a recent finding that the dual topology and dimerization of MRAP2 were regulated by its transmembrane domain [34]. The reversion of the protein sequence retained the capability of MRAP2 proteins to form homo- and heterodimers on the plasma membrane of live cells.

**Figure 3:**
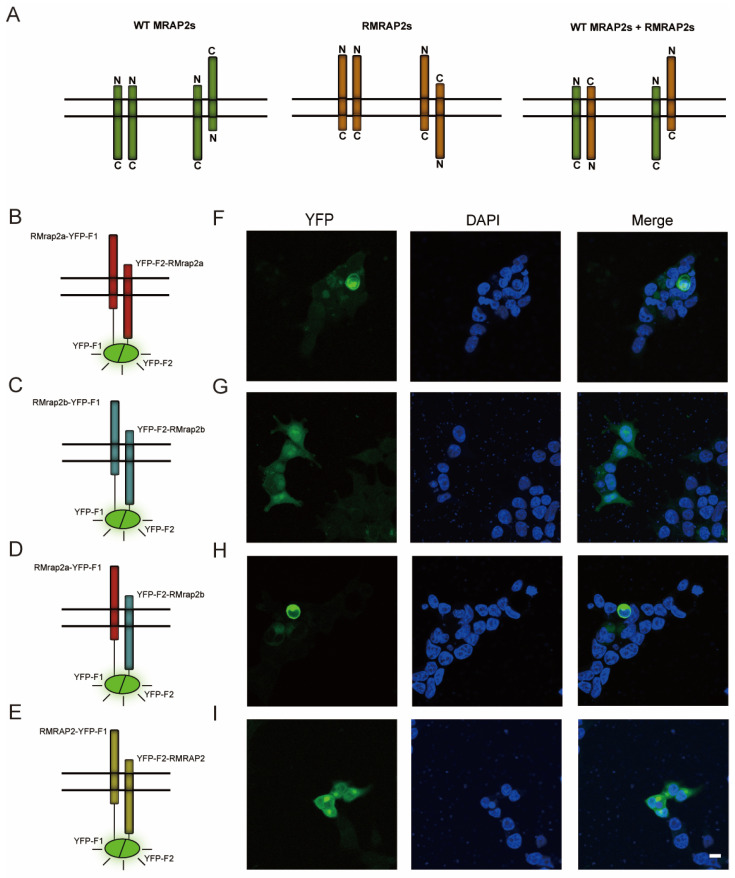
RMrap2a/b and RMRAP2 form antiparallel dimers on the plasma membrane. (**A**) Schematic illustration of parallel and antiparallel dimers of WT MRAP2s (**left**), RMRAP2s (**middle**) as well as WT MRAP2 and RMRAP2 dimers (**right**). (**B**–**E**) The schematic diagrams illustrate the principle of YFP fluorescence emission and the localization of YFP-F1/F2 on the fused protein. Red: RMrap2a, blue: RMrap2b, yellow: RMRAP2. (**F**–**I**) YFP fluorescent and DAPI under confocal microscope. Scale bar = 10 μm.

Theoretically, RMRAP2s should exhibit the same dual topology as WT ones because of the retention of a transmembrane domain, meaning that the dimers could be formed both parallelly and antiparallelly (Figure 3A). To experimentally validate this hypothesis, a bimolecular fluorescent complimentary (BiFC) assay was conducted where YFP protein was separated into YFP-F1 and YFP-F2, and YFP fluorescence could be detected only when these two segments became close enough in live cells. YFP-F1 and YFP-F2 were fused to the N-terminus and C-terminus of MRAP2s, respectively, and co-expressed in the HEK293T cells. The YFP fluorescence indicated the presence of RMrap2a, RMrap2b, RMRAP2 antiparallel homodimers (Figure 3B,C,E–G,I) and RMrap2a/b antiparallel heterodimers (Figure 3D,H). These results suggested that RMRAP2s could be transported to the plasma membrane and existed as dimers bidirectionally just like WT ones (Figure 3A).

### 3.3. RMrap2a and RMrap2b Inhibit the Membrane Trafficking of zMc4r

**Figure 4:**
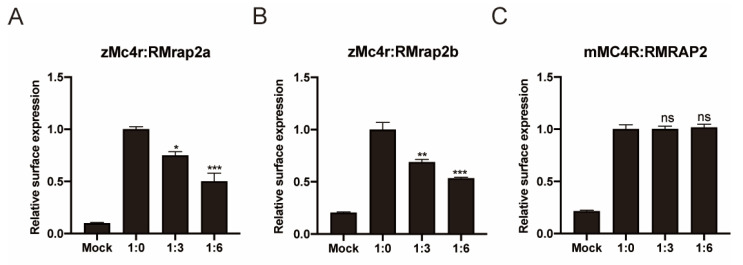
Inhibition of the membrane trafficking of zMc4r by RMrap2a and RMrap2b. (**A**–**C**) The surface expression of zMc4r (**A**,**B**) and mMC4R (**C**) with the increasing dosages of RMrap2a/b and RMRAP2. Mock: blank control transfected with pcDNA3.1. Data were analyzed by one-way ANOVA and are shown as mean ± SEM of three replicates. ns (not significant difference), * *p* < 0.05, ** *p* < 0.01 and *** *p* < 0.001.

Subsequently, the pharmacological function of RMRAP2s was further assessed by examining their assistance to zMc4r and mMC4R signaling. Our previous study showed that Mrap2a and Mrap2b distinctly influenced the surface expression of zMc4r in the zebrafish [36], where zMrap2a decreased the surface expression of zMc4r in a dose-dependent manner, while zMrap2b showed no influence on zMc4r trafficking. Here, we showed that zRMrap2a exhibited a similar trend to zMrap2a (Figure 4A), but zRMrap2b reduced the surface expression of zMc4r by 50%, with the ratio of zMc4r: zRMrap2b = 1:6 (Figure 4B), that differed from WT zMrap2b. In mammals, human MRAP2 decreased the surface expression of MC4R [38], and similar results in mouse MRAP2 were obtained in our previous study. Nevertheless, mRMRAP2 appeared to lose its inhibitory function for mMC4R trafficking (Figure 4C). These distinct results for RMRAP2s were intriguing and suggested that, at least for zMrap2b and mMRAP2, the function of the C-terminus differed from the N-terminus. In addition, we could also speculate that the orientation of MRAP2s (either N-terminus orienting intracellularly or extracellularly) was not random, as there would be no functional difference between zMrap2b and zRMrap2b or mMRAP2 and mRMRAP2 otherwise.

### 3.4. RMrap2a/b Affects Pharmacological Activity of zMc4r

**Figure 5:**
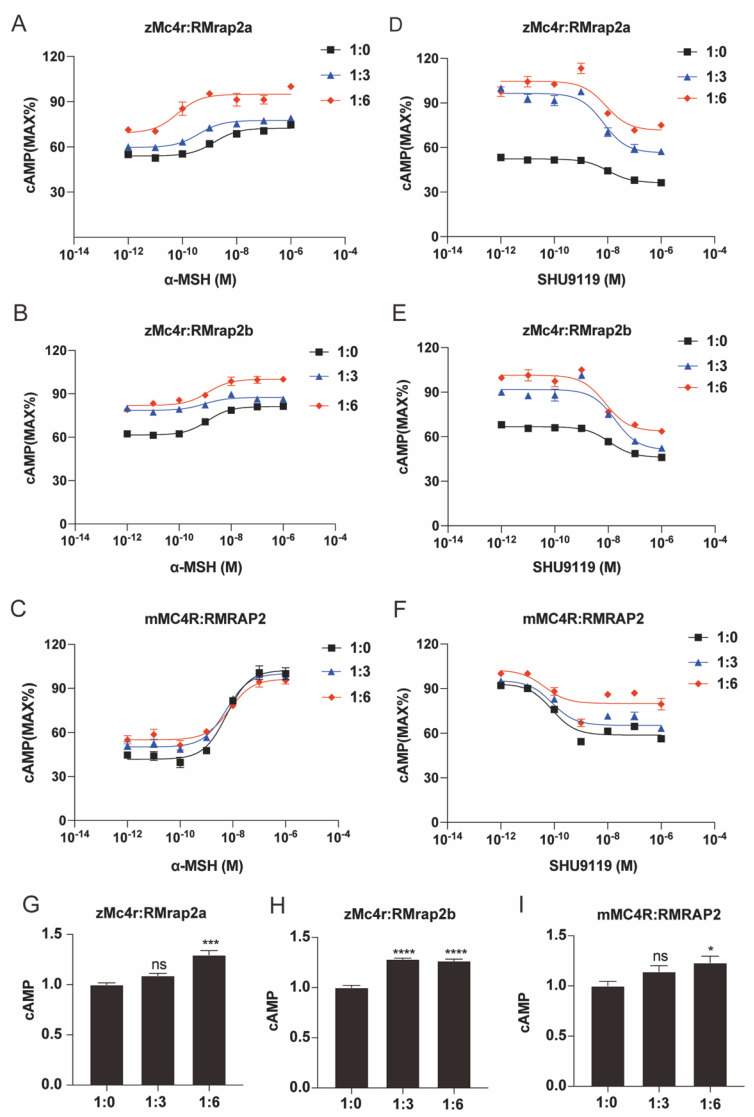
Pharmacological modulation of zMc4r signaling by RMrap2a/b. (**A**–**C**) Dose–response stimulation of MC4R by α−MSH−induced cAMP production in the presence of different amounts of RMrap2a/b (**A**,**B**) or RMRAP2 (**C**). (**D**–**F**) Dose–response inhibition of MC4R by antagonist SHU9119 in the presence of different amounts of RMrap2a/b (**D**,**E**) or RMRAP2 (**F**). (**G**–**I**) The constitutive activity of MC4R in different dosages of RMRAP2. Data were analyzed by one−way ANOVA and shown as mean ± SEM of three replicates. ns (not significant difference), * *p* < 0.05, *** *p* < 0.001 and **** *p* < 0.0001.

The pharmacological activity and intracellular signal transduction of MC4R is significantly influenced by MRAP2. Therefore, it is essential to assess the function of RMRAP2s in this aspect. The common ligands of MC4R include ACTH (agonist), α-MSH (agonist), Agouti-related protein (AgRP) (antagonist) and SHU9119 (antagonist) [39]. The activation of MC4R promoted adenylate cyclase activity and cAMP production [28], so the cAMP level was utilized to reflect the signaling activity of MC4R. In our experiment, zebrafish *mc4r* and *mrap2a* or *mrap2b* were transfected into cells at different ratios (1:0, 1:3, 1:6). Then, these cells were stimulated by α-MSH or suppressed by SHU9119 at different concentrations (10^−12^–10^−6^ M). Different from WT zMrap2a, which inhibited zMc4r action [31], zRMrap2a increased both zMc4r’s sensitivity to α-MSH and its constitutive activity (Figure 5A,D,G and Table 1). As seen in Figure 5B,E,H, zRMrap2b also exhibited a similar function. However, unlike WT mMRAP2, which significantly sensitized mMC4R [28], the sensitizing effect of mRMRAP2 seemed subtle or even nonexistent (Figure 5C,F,I). Overall, both mouse and zebrafish RMRAP2 significantly increased the constitutive activity of MC4R but showed no order of magnitude changes on the EC50 (Table 1 and Table 2). Interestingly, only zebrafish RMRAP2a/b was capable of increasing the maximum response of zMC4R. Our results suggest that the presence of zRMRAP2a and 2b may contribute to the maintenance of long-term energy homeostasis in zebrafish, by enhancing the maximum activity of Mc4r as a tonic satiety signal. This may be a possible mechanism by which zebrafish are more resistant to starvation than mammals such as mice and humans.

**Figure 6:**
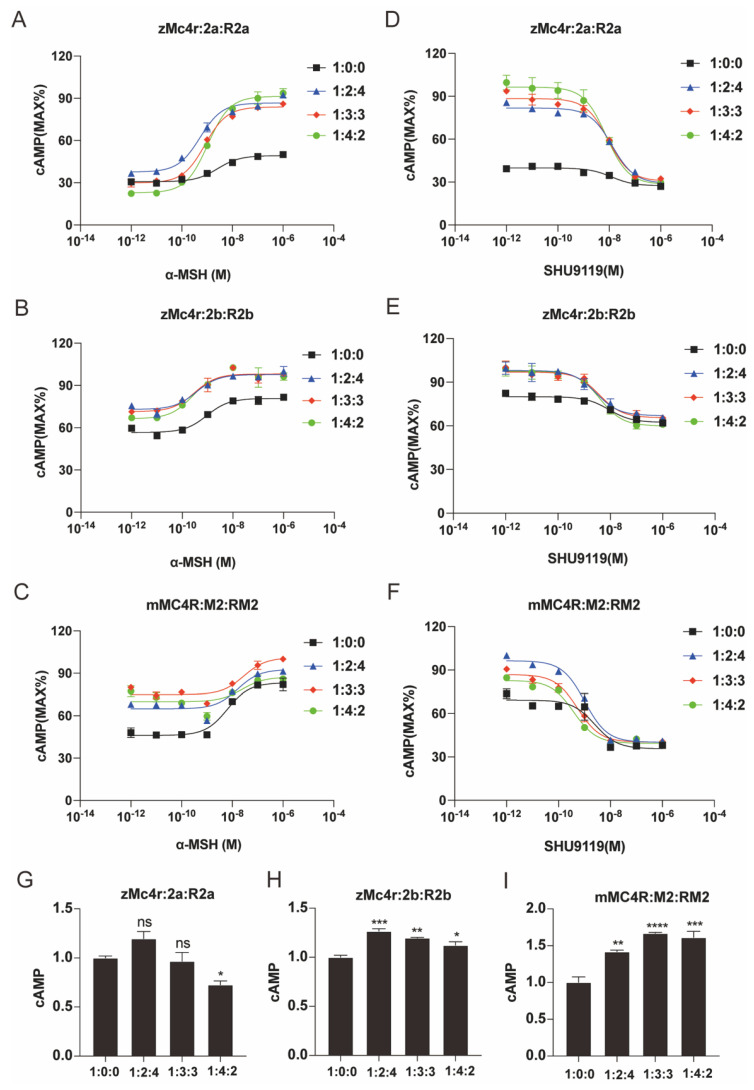
Wild−type and reversed Mrap2a/b differently affect the pharmacological profiles of zMc4r. (**A**–**C**) Dose−responsive stimulation of MC4R by α−MSH−induced cAMP production with different ratios of wild-type and reversed Mrap2a/b (**A**,**B**) or MRAP2 (**C**). (**D**–**F**) Dose−responsive inhibition of MC4R by antagonist SHU9119 with different ratios of wild-type and reversed Mrap2a/b (**D**,**E**) or MRAP2 (**F**). (**G**–**I**) The basal cAMP level caused by transfected MC4R, MRAP2 and RMRAP2 in the absence of an agonist. Data were analyzed by one−way ANOVA and shown as mean ± SEM of three replicates. ns (not significant difference), * *p* < 0.05, ** *p* < 0.01, *** *p* < 0.001 and **** *p* < 0.0001. 2a: Mrap2a, R2a: RMrap2a, 2b: Mrap2b, R2b: RMrap2b, M2: MRAP2, RM2: RMRAP2.

Data shown in Table 1 represent the EC50 of dose–response curves in Figure 5 and Figure 6. Numbers in brackets are with the 95% confidence intervals of nonlinear fittings. One-way ANOVA and Tukey post-tests were employed to measure the significance between the MC4R expression alone group (1:0) and the experimental groups. * *p* < 0.05, ** *p* < 0.01, *** *p* < 0.001.

Data shown in Table 2 summarize the mean of constitutive and maximal activities of MC4Rs in Figure 5 and Figure 6. The numbers of constitutive activity are normalized by dividing the means of 1:0 groups. The values of maximal activity are relative to the respective constitutive activities (max. activity of 1:0 to constitutive activity of 1:0, max. activity of 1:3 to constitutive activity of 1:3). Statistical differences of constitutive activity are labeled in Figure 5 and Figure 6.

### 3.5. The Distinct Effects of Wild-Type and Reversed Mrap2a/b on Pharmacological Activity of zMc4r

The reversion of MRAP2s produces a novel modulator with new functions. It is interesting to investigate how MC4R is affected when WT and reversed MRAP2s coexist. Zebrafish *mc4r* and mouse *Mc4r* were transfected into HEK293T cells along with different ratios of reversed *mrap2a/b* and *Mrap2* (1:0:0, 1:2:4, 1:3:3 and 1:4:2). As indicated in Figure 6, the cAMP responding curve (CRC) indicated that Mc4r became more sensitive to its ligand with the increasing concentration of zMrap2a/RMrap2a, while the basal cAMP level of zMC4R reduced (Figure 6A,D,G), indicating that the sensitizing effect of zRMrap2a dominated despite the blocking effect of zMrap2a. However, the results of zMrap2b suggested equal assistance of zMrap2b and zRMrap2b for zMC4R signaling because of the overlap of the three curves (blue, red and green) (Figure 6B,E,H). Agonist-stimulated cAMP level, induced by MC4R, increased in the presence of mMRAP2 and mRMRAP2 (Figure 6C,F,I). Overall, the coexistence of both conformations of zebrafish MRAP2a affected zMc4r activity, mainly by increasing its maximal activity. However, the simultaneous presence of both conformations of mouse MRAP2 affected mMC4R activity mainly by enhancing its basal activity. Additionally, the simultaneous presence of two orientations of zebrafish MRAP2b enhanced both the constitutive activity and maximal activity of zMC4R (Table 1 and Table 2).

## 4. Discussion

As a key member of the MCR family, MC4R plays crucial roles in regulating energy homeostasis and feeding behavior. Therefore, MC4R has become a promising GPCR drug target for severe obesity therapy. Setmelanotide, synthesized as an agonist for MC4R, was approved by the U.S. Food and Drug Administration (FDA) in the United States in November 2020, and in the European Union in July 2021, for alleviating severe obesity syndrome. Because of the significant sensitization of MC4R cascades by MRAP2 proteins, it is plausible to enhance the agonist stimulation through conformational allosteric modulation. Therefore, it is meaningful and valuable to explore the regulatory mechanism of MRAP2 for the modulation of MC4R signaling. Functional MRAP2 complex exists on the plasma membrane as dimers or oligomers. Given the presence of antiparallel homodimers of MRAP2, we want to uncover the difference between two orientations of MRAP2, either the N-terminus orientating intracellularly or extracellularly. To test this hypothesis, zebrafish RMrap2a, zebrafish RMrap2b and mouse RMRAP2 were synthesized and the dimerization, protein interaction and pharmacological activities on MC4R signaling were assessed in this study.

It has been reported that reversed protein sequences are perhaps more foldable than native ones and the ability of reversed sequences to adopt native-like folds is strongly influenced by protein size and the flexibility of the native hydrophobic core [40]. MRAP2 functions as a short and small protein with only a α-helix in the secondary structure; the reversion of MRAP2, theoretically, will not affect its normal folding and our results demonstrate that all three RMRAPs could express and be transferred normally to the cell surface. The proper interaction with MC4R and dimerization indicates their native-like folding capability, just like the native ones. In addition, they could also interplay with their respective WTs to form novel dimeric complexes that might exert distinctly regulatory functions. Some hints have emerged of the effect of these novel MRAP2 and RMRAP2 complexes on the pharmacological modulation of MC4R signaling in our analysis. However, it accurately defining these functions remains elusive since it is hard to determine what forms prevail with the coexistence of MRAP2s and RMRAP2s in the same cell (Figure 3A).

Despite their native-like structure, mouse and zebrafish RMRAP2s differ from WTs in the regulation of MC4R trafficking and signaling. RMrap2a shows elevating regulation of Mc4r signaling, which is opposite to the effect of Mrap2a [31]. Unlike WT Mrap2b, which has no effect on the trafficking, RMrap2b down-regulates Mc4r trafficking process [36]. In contrast, MRAP2’s influence in modulating mouse MC4R translocation is deprived upon sequence reversion. The different results for zebrafish and mice imply that mammalian MRAP2 proteins are more susceptible to artificial modification. Perhaps some protein families have been eliminated due to redundancy over the course of evolution; therefore, the mammalian proteomes are too precise to withstand unexpected alteration.

Combining the results of surface translocation and pharmacological modulation, it can be proven that different parts of MRAP2 own different functions, and the domain for assisting MC4R trafficking and the one influencing MC4R signaling are in distinct locations. This finding is similar to the analysis concluded by Rouault et al. [21] in which they located the region of MRAP2 protein required for the signaling and trafficking of orexin and prokineticin receptor. It can be further inferred that the N- and C-terminus of Mrap2a show the same effect on regulating Mc4r trafficking but perform opposite roles on downstream MC4R signaling. For Mrap2b, either the N- or C-terminus affected Mc4r trafficking, but both terminuses increased Mc4r signaling. In contrast, RMRAP2 lost its assisting role for MC4R, although it could still be properly expressed, folded and dimerized.

When MRAP2s and RMRAP2s were co-expressed in the same cells, we found that the sensitizing effect of RMrap2a dominated despite the presence of the blocking effect of Mrap2a. In contrast, Mrap2b and RMrap2b showed equal assistance for MC4R signaling. The opposite modulation of Mrap2a’s N-terminus and C-terminus in MC4R signaling was a hint for identifying precise motif enhancing or blocking Mc4r signal transduction.

In conclusion, our results confirmed the symmetry of the MRAP2 transmembrane domain and revealed the non-random orientation of MRAP2 proteins. Surface expression and CRC assay identified reversed Mrap2a and Mrap2b as new functional modulators, exerting a novel impact on regulating Mc4r trafficking and signaling. These findings will help us to further understand the asymmetry of the opposite ends of MRAP2 and its dual orientation on the plasma membrane, and may provide scientific inspiration for how to create a novel accessory and allosteric modulator for melanocortin receptors or other GPCRs in the future.

## Figures and Tables

**Table 1:** Pharmacological summary for logEC50 of dose–response curves in Figure 5 and Figure 6.

		EC50			
Figure 5		1:0	1:3	1:6	
A	zMc4r:RMrap2a (α-MSH)	1.51 × 10^−9^ [±1.06 × 10^−9^]	4.01 × 10^−10 ns^ [±2.49 × 10^−10^]	6.62 × 10^−11^ *** [±9.93 × 10^−11^]	
B	zMc4r:RMrap2b (α-MSH)	1.23 × 10^−9^ [±0.55 × 10^−9^]	9.42 × 10^−10^ * [±11.80 × 10^−10^]	1.28 × 10^−9^ *** [±1.56 × 10^−9^]	
C	mMC4R:RMRAP2 (α-MSH)	5.79 × 10^−9^ [±2.83 × 10^−9^]	5.47 × 10^−9 ns^ [±2.06 × 10^−9^]	7.46 × 10^−9 ns^ [±5.43 × 10^−9^]	
D	zMc4r:RMrap2a (SHU9119)	1.04 × 10^−8^ [±0.67 × 10^−8^]	6.97 × 10^−9^ *** [±5.21 × 10^−9^]	8.59 × 10^−9^ *** [±10.16 × 10^−9^]	
E	zMc4r:RMrap2b (SHU9119)	1.11 × 10^−8^ [±0.75 × 10^−8^]	1.80 × 10^−8 ns^ [±1.74 × 10^−8^]	7.68 × 10^−9^ ** [±6.31 × 10^−9^]	
F	mMC4R:RMRAP2 (SHU9119)	8.17 × 10^−11^ [±6.02 × 10^−11^]	8.37 × 10^−11 ns^ [±11.60 × 10^−11^]	4.20 × 10^−11 ns^ [±11.56 × 10^−11^]	
Figure 6		1:0:0	1:2:4	1:3:3	1:4:2
A	zMc4r:Mrap2a:RMrap2a (α-MSH)	2.60 × 10^−9^ [±1.74 × 10^−9^]	5.26 × 10^−10 ns^ [±3.01 × 10^−10^]	8.17 × 10^−10 ns^ [±2.61 × 10^−10^]	1.07 × 10^−9 ns^ [±0.37 × 10^−9^]
B	zMc4r:Mrap2b:RMrap2b (α-MSH)	9.44 × 10^−10^ [±6.12 × 10^−10^]	3.60 × 10^−10^ * [±4.00 × 10^−10^]	3.02 × 10^−10^ * [±3.73 × 10^−10^]	2.50 × 10^−10^ * [±3.02 × 10^−9^]
C	mMC4R:MRAP2:RMRAP2 (α-MSH)	6.48 × 10^−9^ [±4.15 × 10^−9^]	1.62 × 10^−8 ns^ [±1.91 × 10^−8^]	2.81 × 10^−8^ ** [±3.43 × 10^−8^]	2.41 × 10^−8 ns^ [±12.62 × 10^−8^]
D	zMc4r:Mrap2a:RMrap2a (SHU9119)	1.23 × 10^−8^ [±1.42 × 10^−8^]	1.30 × 10^−8 ns^ [±0.55 × 10^−8^]	9.03 × 10^−9^ * [±3.97 × 10^−9^]	7.53 × 10^−9^ * [±3.16 × 10^−9^]
E	zMc4r:Mrap2b:RMrap2b (SHU9119)	8.78 × 10^−9^ [±11.20 × 10^−9^]	2.87 × 10^−9^ *** [±4.11 × 10^−9^]	3.85 × 10^−9^ *** [±3.40 × 10^−9^]	3.99 × 10^−9^ *** [±2.01 × 10^−9^]
F	mMC4R:MRAP2:RMRAP2 (SHU9119)	2.49 × 10^−9^ [±3.05 × 10^−9^]	1.02 × 10^−9 ns^ [±0.39 × 10^−9^]	5.47 × 10^−10 ns^ [±2.29 × 10^−10^]	3.83 × 10^−10 ns^ [±1.37 × 10^−10^]

Data shown in Table 1 represent the EC50 of dose–response curves in Figure 5 and Figure 6. Numbers in brackets are with the 95% confidence intervals of nonlinear fittings. One-way ANOVA and Tukey post-tests were employed to measure the significance between the MC4R expression alone group (1:0) and the experimental groups. ns (not significant difference), * *p* < 0.05, ** *p* < 0.01, *** *p* < 0.001.

**Table 2:** Summary of the constitutive and maximal activities of MC4Rs in Figure 5 and Figure 6.

Figure 5			1:0	1:3	1:6	
A	zMc4r:RMrap2a	constitutive activity	100%	109.1%	129.7%	
		maximal activity	134.1%	130.0%	137.1%	
B	zMc4r:RMrap2b	constitutive activity	100%	128.3%	126.7%	
		maximal activity	132.0%	111.4%	122.1%	
C	mMC4R:RMRAP2	constitutive activity	100%	114.2%	123.1%	
		maximal activity	245.2%	199.4%	175.1%	
Figure 6			1:0:0	1:2:4	1:3:3	1:4:2
A	zMc4r:Mrap2a:RMrap2a	constitutive activity	100%	119.7%	96.7%	72.7%
		maximal activity	160.3%	230.5%	280.3%	400%
B	zMc4r:Mrap2b:RMrap2b	constitutive activity	100%	126.7%	119.7%	112.3%
		maximal activity	142.6%	133.9%	137.5%	147.8%
C	mMC4R:MRAP2:RMRAP2	constitutive activity	100%	142.0%	167.0%	161.3%
		maximal activity	180.6%	142.8%	134.8%	124.7%

Data shown in Table 2 summarize the mean of constitutive and maximal activities of MC4Rs in Figure 5 and Figure 6. The numbers of constitutive activity are normalized by dividing the means of 1:0 groups. The values of maximal activity are relative to the respective constitutive activities (max. activity of 1:0 to constitutive activity of 1:0, max. activity of 1:3 to constitutive activity of 1:3). Statistical differences of constitutive activity are labeled in Figure 5 and Figure 6.

## Data Availability

All of data generated from this study are included in the article.

## Supplementary Materials

The following supporting information can be downloaded at: https://www.mdpi.com/article/10.3390/biology11060874/s1. Figure S1: (**A**–**C**) TM regions of RMrap2a (**A**), RMrap2b (**B**) and RMRAP2 (**C**). The dense red vertical lines indicate TM regions, and the blue and purple lines represent different orientations of N- and C-terminus, as well as their respective possibility score. Figure S2: Intensity ratio of Western blot bands in Figure 1 and Figure 2. (**A**–**D**) Intensity ratio of each band corresponding to panel (**C**–**F**) in Figure 1. (**E**–**K**) Intensity ratio of each band corresponding to panel (**A**–**G**) in Figure 2.
