# Supplementary material for: Reversion of MRAP2 Protein Sequence Generates a Functional Novel Pharmacological Modulator for MC4R Signaling
Source: Biology (Basel). 2022 Jun 7;11(6):874. doi: 10.3390/biology11060874 (PMC9219869; doi:10.3390/biology11060874)
Supplement: Supplementary file 1 [file biology-11-00874-s001.zip › biology-1696266-supplementary.pdf]

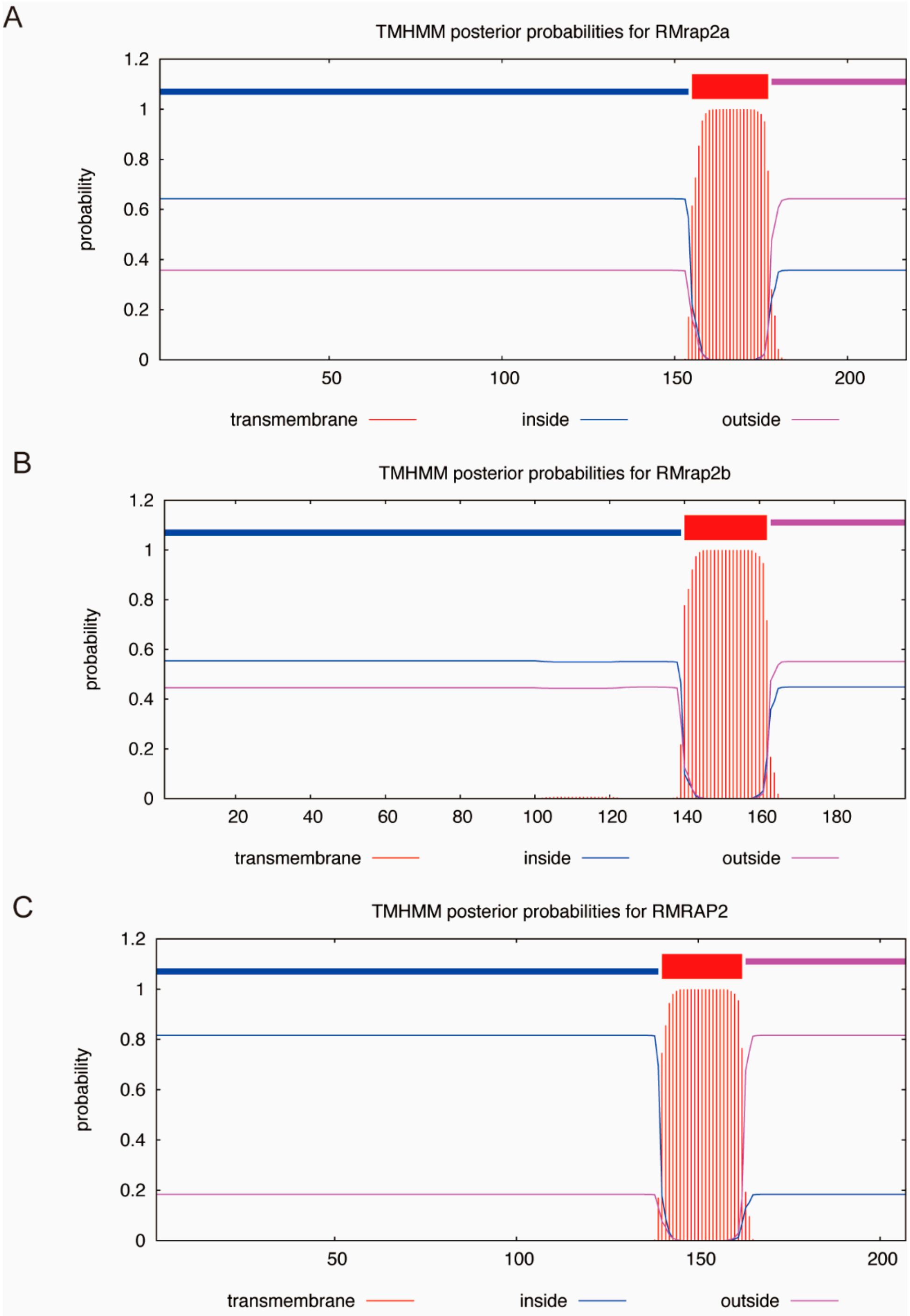

Supplementary Figure S1. TM domain prediction of RMRAP2s

(**A**–**C**) TM regions of RMrap2a (**A**), RMrap2b (**B**) and RMRAP2 (**C**). The dense red vertical lines indicate TM regions, and the blue and purple lines represent different orientations of N- and C-terminus, as well as their respective possibility score.

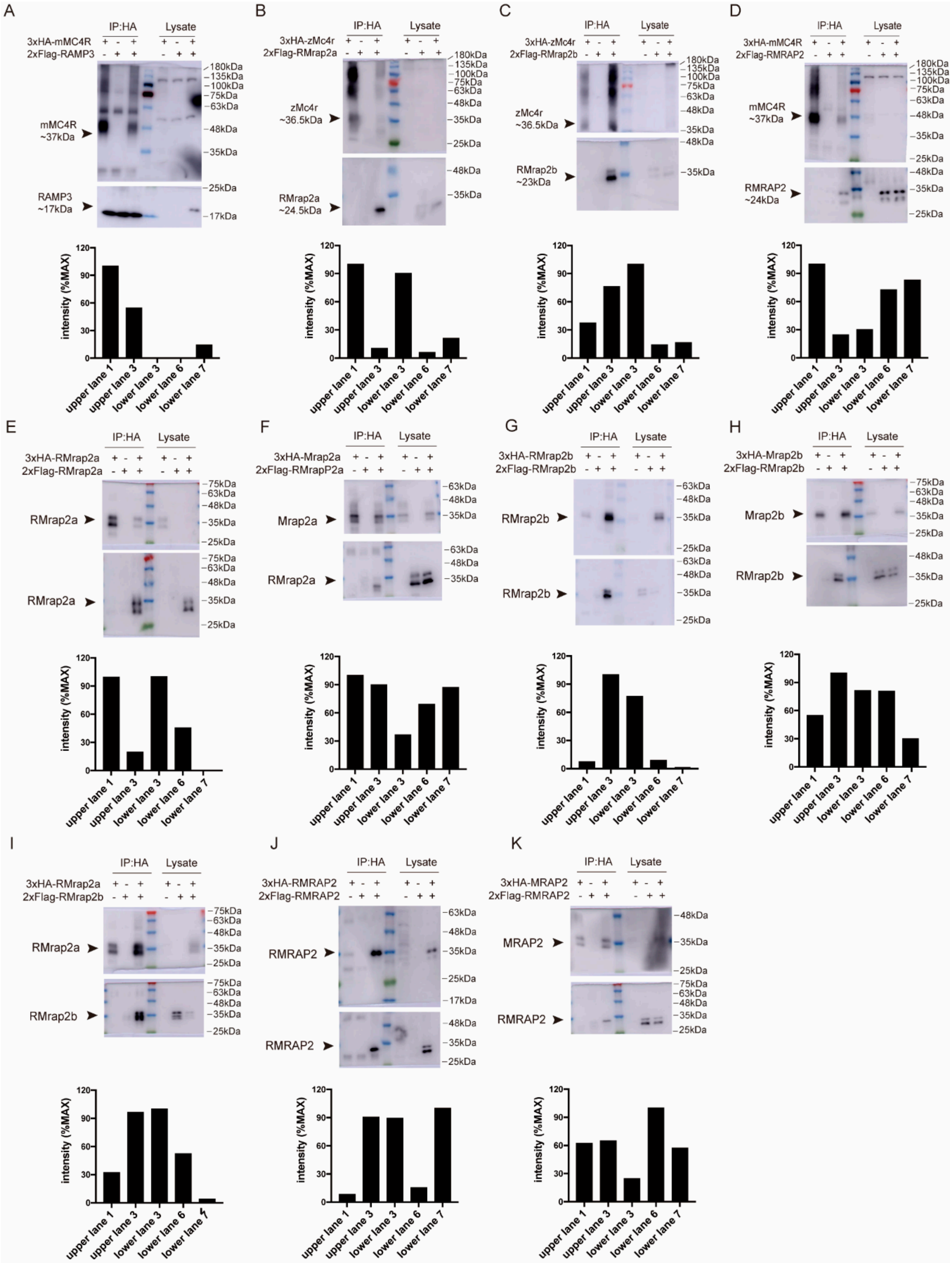

Supplementary Figure S2. Intensity ratio of Western blot bands in Figure 1 and Figure 2. (**A–D**) The whole blot and relevant intensity ratio of each band corresponding to panel (**C–F**) in Figure 1. (**E–K**) The whole blot and relevant intensity ratio of each band corresponding to panel (**A–G**) in Figure 2.
